# Time-Course Analysis of Main Markers of Primary Infection in Cats with the Feline Immunodeficiency Virus

**DOI:** 10.1155/2012/342602

**Published:** 2012-09-25

**Authors:** B. Ribba, H. El Garch, S. Brunet, E. Grenier, F. Castiglione, H. Poulet, P. Vanhems

**Affiliations:** ^1^INRIA, Project-team NUMED, Ecole Normale Supérieure de Lyon, 46 allée d'Italie, 69007 Lyon Cedex 07, France; ^2^Discovery Research, Merial SAS, 69007 Lyon, France; ^3^Institute for Computing Applications “M. Picone”, National Research Council of Italy (CNR), Rome, Italy; ^4^Service d'Hygiène, Epidémiologie et Prévention, Hospices Civils de Lyon, 69008 Lyon, France; ^5^Université de Lyon, 69000 Lyon, Lyon, Université Lyon I, Villeurbanne, F-69100, France; ^6^Université de Lyon I, 69000 Villeurbanne, France; ^7^Equipe Epidémiologie et Santé Publique, Laboratoire de Biométrie et Biologie Evolutive, UMR 5558, CNRS, 8 Avenue Rockefeller, 69310 Lyon cedex, France

## Abstract

Studies of the response of the immune system to feline immunodeficiency virus (FIV) during primary infection have shown that a subpopulation of CD8^+^ T-cells with an activated phenotype and reduced expression of the CD8*β* chain (denoted CD8*β* ^low^ T cells) expands to reach up to 80% of the total CD8^+^ T cell count. The expansion of this subpopulation is considered to be a signature of FIV and an indicator of immune system alteration. We use a simple mathematical formalism to study the relationships over time between the dose of infection, the size of the CD8*β* ^low^ population, and the circulating viral load in cats infected with FIV. Viremia profiles are described using a combination of two exponential laws, whereas the CD8*β* ^low^ percentage (out of the total CD8^+^ population) is represented by a Gompertz law including an expansion phase and a saturation phase. Model parameters are estimated with a population approach using data from 102 experimentally infected cats. We examine the dose of infection as a potential covariate of parameters. We find that the rates of increase of viral load and of CD8*β* ^low^ percentage are both correlated with the dose of infection. Cats that develop strong acute viremia also show the largest degree of CD8*β* ^low^ expansion. The two simple models are robust tools for analysing the time course of CD8*β* ^low^ percentage and circulating viral load in FIV-infected cats and may be useful for generating new insights on the disease and on the design of therapeutic strategies, potentially applicable to HIV infection.

## 1. Introduction

Cats infected with the feline immunodeficiency virus (FIV) develop an acquired immunodeficiency syndrome (AIDS) much like humans infected with HIV [1]. The infection causes an acute viremia, which decreases after several weeks, and the development of a partial immunity [2]. The acute stage is followed by a chronic asymptomatic phase, often persisting for years, during which the immune system is progressively impaired. As in the case of HIV infection, the more common signs of the asymptomatic phase are the depletion of CD4^+^ peripheral T cells and the reduction of the CD4/CD8 ratio [3]. At the end of the asymptomatic stage of the disease, infected cats develop chronic opportunistic infections and eventually die [4]. 

The immune response to FIV during acute infection is well documented in the literature (see in particular [5–7]). In addition to anti-FIV neutralizing antibodies and cytotoxic and noncytotoxic CD8^+^ T cells, the primary (acute) stage of infection is known to be characterized by the appearance and expansion of a CD8^+^ T-cell subpopulation with an activated phenotype showing reduced expression of the CD8*β* chain and the complete disappearance of the L-selectin CD62L surface molecule [8]. These CD8*β* ^low^ CD62L^−^ T cells, hereafter referred to as CD8*β* ^low^ cells, persist throughout the course of infection. The persistence of this activated T-cell population, which has been shown to possess anti-FIV activity, suggests a profound homeostatic disorder, as in healthy animals activated CD8 cells are generally present only during peak immune responses.

The observed expansion of the CD8*β* ^low^ cell subpopulation—which can reach, during the acute stage, up to 80% of the total population of CD8^+^ T cells—may be driven by CD8*β* ^low^ cells' sensitivity to apoptosis, a sensitivity that is enhanced by antigen recognition [2]. CD8*β* ^low^ cells might be chronically activated as a result of persistent virus replication and antigen recognition, die by apoptosis and get replenished quickly. Thus, it is believed that FIV can alter the immune homeostasis by inducing chronic activation of CD8^+^ T cells into CD8*β* ^low^, driving their expansion, and, at the same time, inducing cytotoxicity against infected CD4^+^ T cells. 

The expansion of the CD8*β* ^low^ subpopulation is considered to be an important marker of FIV infection and disease [2]. A characterization of the process of expansion, in addition to other markers of disease, is likely to increase researchers' understanding of FIV infection and AIDS pathogenesis, thus facilitating the design of new therapeutic strategies. 

Mathematical models to describe longitudinal data from HIV-infected patients have been extensively developed [9]. To describe the dynamics of viremia and CD4^+^ T cells, numerous models have used systems of ordinary differential equations based on the prey-predator modelling framework [10–13]. In this type of model, viral particles infect healthy CD4^+^ T cells, which later die, liberating new replicated virus into the plasma. One of the most interesting aspects of this mechanistic approach is that each model parameter has a clear biological meaning, such as rate of infection of CD4^+^ T cells, the cell lifespan, or the virus replication rate. Such models have been shown to correctly predict circulating viral loads in HIV-infected patients undergoing antiviral therapies [11]. Some variations of this modelling approach have been discussed in the literature. For instance, the integration of cytotoxic CD8^+^ T cells has been shown to potentially describe with more accuracy the kinetics of viremia in HIV patients [14]. Models based on the prey-predator framework can provide interesting insights into the life cycle of the virus and its interaction with the host. However, these models tend to be complex, as they generally integrate a large number of parameters and variables such as viral load, CD4^+^ T cells of different status (e.g., uninfected, early-stage infected, late-stage infected), and different types of CD8^+^ T cells. Proper estimation of such parameters requires a large number of observations (ideally, observations for all variables should be available) for all individuals to be analyzed. Obviously, these are difficult conditions to meet in a clinical setting. Furthermore, it is known that the immune response can vary significantly across subjects, and it might therefore be too simplistic to assume parameters to be constant in a given population of patients. The need to integrate interindividual results adds an additional level of complexity to the already complex mechanistic model. Finally, to our knowledge, such models have not yet been challenged with data from untreated primary infection (e.g., data from untreated HIV-infected patients or from FIV-infected cats), so the information they provide regarding the natural progression of disease may be limited.

In this study, we propose two phenomenological models that correctly reproduce the time-evolution of the percentage of CD8*β* ^low^CD62L^−^ T cells and of circulating viral load during the early primary infection phase in 102 cats infected with various doses of FIV.

## 2. Materials and Methods

### 2.1. Ethic Statement

All animal experiments were conducted in accordance with the European Community regulations, and all procedures were supervised and approved by the Merial Ethical Committee.

### 2.2. Animals

In this experiment, 102 cats (49 males and 53 females; mean age: 22.8 weeks, SD: 7.7, range: 13–36.5) were randomized into 23 groups of 4 to 7 cats each. Each group was assigned an FIV strain (Petaluma clade A, Glasgow-8 clade A, or EVA clade B) and inoculum size. Each cat was challenged with a single intramuscular injection of 1 mL of viral suspension of one of the three FIV strains examined. In preliminary *in vitro* experiments, the three strains were observed to be comparable in terms of viremia and impact on lymphocyte subpopulations. Virus dilutions ranged from 1/90,000 to 1/3, and the infection doses, expressed in log_10_/mL of cell culture infectious dose 50% (CCID50), ranged from 0.26 to 4.09 (median: 2.5, SD: 1.21). 

### 2.3. Longitudinal Measurements

Viral load was measured using quantitative real-time polymerase chain reaction. For each cat, a measurement was taken at time 0, and, when possible, additional measurements were taken at the ends of weeks 1, 3, 4, 6, 9, 12, 15, 18, and 23. Values were expressed as log_10_ of viral RNA copies per millilitre of plasma. For these measurements, the detection threshold, or the limit of quantification (LOQ), was 80 copies per mL, which corresponds to 1.9 on the log_10_ scale. In total, 485 measurements were analyzed, but there was high variability in the number of measurements per cat (mean = 4.75 measurements/cat, min = 1, max = 7, SD = 1.2). The values themselves (all taken together) were also highly variable (median = 3.95 log_10_ RNA copies/mL of plasma, min = 1.9 (LOQ), max = 6.91, SD = 1.37).

**Figure 1:**
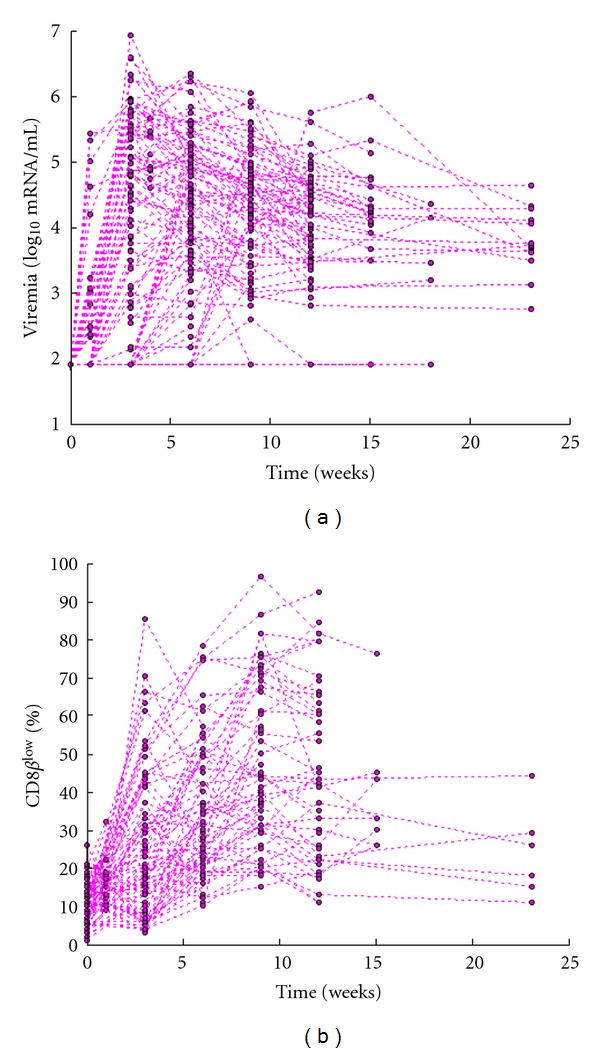
Time-evolution of circulating viral load, expressed as log_10_ of viral RNA copies per mL plasma (a); CD8*β* ^low^ population size expressed as the percentage of the CD8*β* ^low^CD62L^−^ T-cell subpopulation out of the total population of CD8^+^ T cells (b). Time is expressed as weeks after infection. Viral load was measured in 102 cats, but CD8*β* ^low^ cell counts were measured in only 79 cats.

The number of CD8*β* ^low^ cells and the total number of CD8^+^ T cells were measured by flow cytometry as described in [2]. However, data on these lymphocytes were available for only 79 cats out of the total 102. The size of the CD8*β* ^low^CD62L^−^ subpopulation was expressed as the percentage of CD8*β* ^low^CD62L^−^ T cells in the entire CD8^+^ T-cell population. The analysis was carried out on 377 observations with an average of 4.8 observations per animal (min = 3, max = 6, SD = 0.8). The median observed value of CD8*β* ^low^ percentage was 22% (min = 1%, max = 96%, SD = 20.5).  Figure 1 shows the time-evolution of viral load in all 102 cats (Figure 1(a)) and CD8*β* ^low^ percentage in the 79 cats (Figure 1(b)) for which lymphocyte counts were available. The curves indicate high variability across cats in both viral load and CD8*β* ^low^ percentages.

### 2.4. Data Analysis

The high variability in the number of available data points per animal, as well as the variability across animals in the patterns of the data, required the use of mixed-effects regression techniques. Mixed-effects models take into account different forms of variability and, in particular, interindividual variability [15]. More precisely, they use the available information from all individuals of an analyzed population to retrieve both population-level and individual-level values for the dynamic parameters. As a consequence, they are particularly suited for the analysis of datasets with large numbers of individuals, even if data are sparse for some of the individuals.

In their general form, such models can be written as follows:

(1) $$\begin{array}{c} y_{ij}=f\left(x_{ij},\phi_{i}\right)+g\left(x_{ij},\phi_{i}\right)\varepsilon_{ij},\;1\leq i\leq N;\;\;1\leq j\leq n_{i}, \end{array}$$

where *N* is the number of animals, *n*_*i*_ the number of observations for individual *i*, *x* the regression variable (e.g., time), and *y* the observations. The term *f* represents deterministic equations; in our case, these are simple phenomenological laws. The residual error is *g*(*x*_*ij*_, *ϕ*_*i*_)*ε*_*ij*_, where *ε*_*ij*_ ~ *N*(0, *σ* ^2^). In what follows we will consider constant error models, that is, *g*(*x*_*ij*_, *ϕ*_*i*_) = 1.

Each individual parameter *ϕ*_*i*_ can be defined as follows:

(2) $$\begin{array}{c} \phi_{i}=h\left(\mu +\eta_{i}\right),\;\eta_{i}\sim N\left(0,\Omega \right),\;\;i=1,\ldots ,N, \end{array}$$

where *η*_*i*_ is a *p*-vector of random effects and *h* is some predefined transformation. Here, we assume that the individual parameters are log-normally distributed (i.e., *h*(*u*) = *e* ^*u*^). *μ* is a *p*-vector of fixed population parameters (i.e., *h*(*μ*) is the median value across individuals for each of the *p* parameters). *Ω* is the *p* × *p* variance-covariance matrix of the random effects. We assume potential correlations between the random effects, meaning that *Ω* is a full matrix.

The unknown set of parameters in the model is then

(3) $$\begin{array}{c} \theta =\left(\mu ,\Omega ,\sigma^{2}\right). \end{array}$$

The likelihood function related to this problem can be written as follows:

(4) $$\begin{array}{c} L\left(\theta ,y\right)=\prod_{i=1}^{N}L_{i}\left(\theta ,y_{i}\right), \end{array}$$

with

(5) Li(θ,yi)=∫p(yi,ηi,θ)dηi=C∫σ−ni|Ω|−1/2e−1/(2σ2)||yi−f(xi,ϕi)||2−(1/2)ηi′Ω−1ηidηi.

If *f* is nonlinear with respect to the random effects, the likelihood function cannot be easily computed and maximized. One intuitive means of addressing this problem is to analyze the data from each individual separately. This approach, however, requires a large number of observations per individual, and therefore it is clearly not feasible in our case. An alternative method is the SAEM algorithm (stochastic approximation of the EM algorithm [16]), which can be used to calculate the maximum likelihood, without any approximation of the likelihood function and to estimate population (*θ*) and individual (*ϕ*_*i*_) parameters. We used Monolix software (Lixoft) to estimate those parameters. The software analyzes all individual data simultaneously. In a first step, a likelihood function is minimized in order to estimate the mean values of the model parameters as well as their variability throughout the population. The resultant estimates are referred to as the population parameters. In a second step, information on the mean parameter values is used to estimate, on the basis of each individual dataset, the best model parameters for each individual. These are called individual parameters.

Mixed-effects models also have the advantage of being associated with a large panel of validation tools. The log-likelihood (LLH) value (actually −2 × LLH) is generally used to select the best model from among multiple models. However, since a model with a greater number of parameters is more likely to produce a better fit because it has more degrees of freedom, a penalty term is generally added to the likelihood function to account for the number of parameters. Examples of criterion functions that include such penalty terms are the Akaike information criterion (AIC):

(6) $$\begin{array}{c} \text{AIC}=-2\times \text{LLH}\;+2\times n, \end{array}$$

where *n* is the number of free parameters to be estimated, and the Bayesian information criterion (BIC):

(7) $$\begin{array}{c} \text{BIC}=-2\times \text{LLH}+\log\left(k\right)\times n, \end{array}$$

where *k* is the sample size. 

We tested different types of phenomenological models, and we selected the best ones on the basis of three criterion functions—namely, −2 × LLH, AIC, and BIC values— goodness of fit, residual plots, and precision of parameter estimates as relative standard errors. We assessed simulation-based diagnostics through visual predictive check, that is, we graphically compared the observed data and the simulated data (using population parameters and both interindividual and residual variability). We calculated *ε*-shrinkage and *η*-shrinkage to evaluate the degree of shrinkage of individual predictions towards the observations [17]. High values of shrinkage (>30%) are considered to impair diagnostics based on individual predictions and covariate analysis [17].

## 3. Results

### 3.1. Modelling Viremia

**Figure 2:**
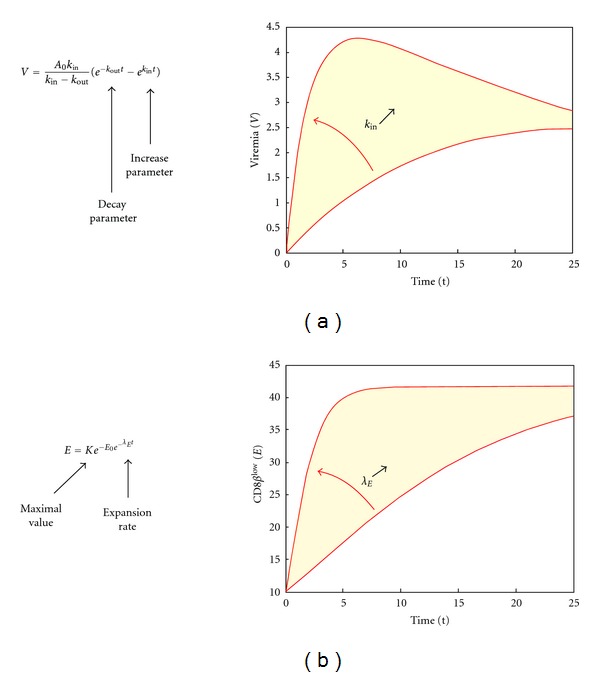
Schematic view and basic simulations of selected models for viremia (a) and CD8*β* ^low^ percentage (b). We highlight here the role of the parameters regulating the increase of viremia and CD8*β* ^low^, respectively.

We first formulated a model to describe the observed pattern of acute increase in viral load followed by decay, as shown in Figure 1(a). The best model we identified was a sum of two exponentials, describing, respectively, the growth and decay parts of the curves:

(8) $$\begin{array}{c} V=\frac{A_{0}k_{\text{in}}}{k_{\text{in}}-k_{\text{out}}}\left(e^{-k_{\text{out}}t}-e^{-k_{\text{in}}t}\right), \end{array}$$

where *V* is the viral load, expressed as log_10_ of the number of viral RNA copies per mL of plasma; *k*_in_ and *k*_out_ are the two parameters regulating, respectively, the increase and decay of viral load; *A*_0_ is a scaling adimensional parameter.  Figure 2(a) shows a schematic view and focuses on the effect of changing the value of the parameter *k*_in_. The higher the parameter value, the more rapid the increase in viral load. Interanimal variability in the model parameters (*A*_0_, *k*_in_, *k*_out_) was assumed to be log-normally distributed, and cat-specific estimates are given as follows, for example, for *k*_in_:

(9) $$\begin{array}{c} k_{\text{i}{\text{n}}_{i}}=k_{\text{in}}e^{\eta_{i}^{k_{\text{in}}}}, \end{array}$$

where *k*_in_ is the typical value for the population (mean value) and *η*_*i*_^*k*_in_^ is an inter-animal random effect that follows a normal distribution with mean 0 and variance *ω*_*k*_in__^2^.

In a second step, the dose of infection and the virus strain were evaluated as continuous and, respectively, categorical covariates. We used a backward-stepwise method to test how inclusion of these covariates affected the three model parameters [18]. Virus strain had no significant effect on the values of any of the three parameters, whereas dose of infection, expressed in log_10_/mL of CCID50, affected the constant rate of increase of viral load. Dose of infection was successfully integrated into *k*_in_, which can be written as follows:

(10) $$\begin{array}{c} k_{\text{in}}=k_{\text{in}}e^{\beta_{k_{\text{in}}}\times \;\;\text{DOSE}}. \end{array}$$

With this covariate integration, the objective function (−2 × LLH) was reduced by 58 points, the parameter *β*_*k*_in__was estimated with high precision (*P* < 0.001), and the variability on the *k*_in_ parameter decreased by 30%.

**Figure 3:**
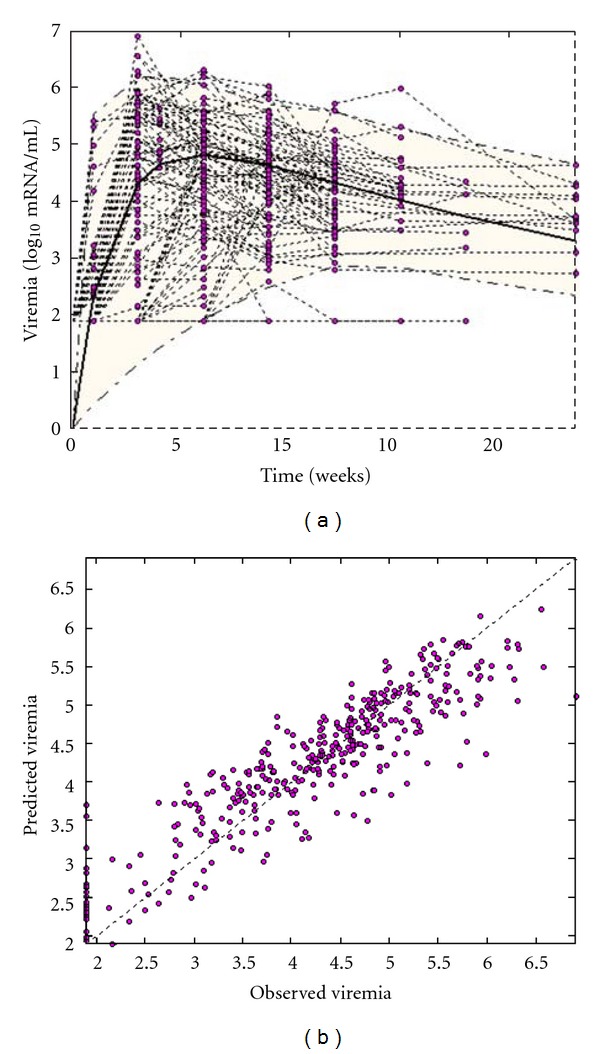
Viremia model diagnostics. (a): Simulation of the population model with 95% of variability together with the data points (visual predictive check). (b): Individual predictions versus the actual observations.

**Figure 4:**
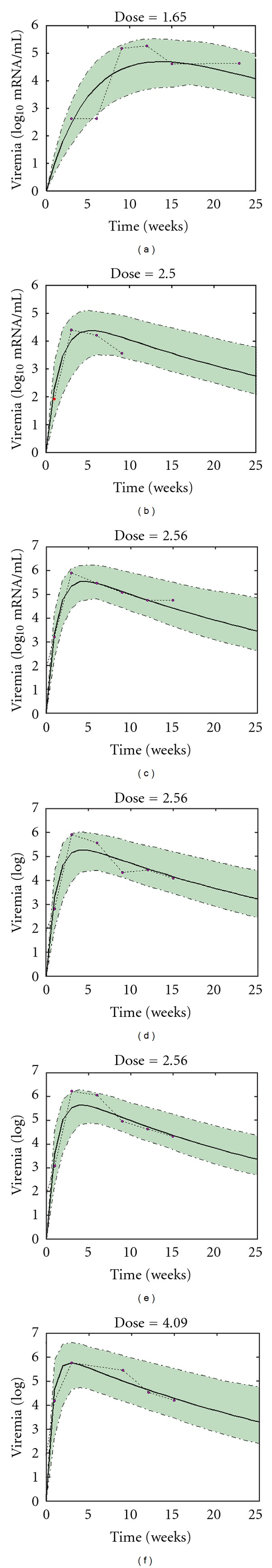
Goodness of individual predictions of viral load. A 95% confidence interval around the prediction for six cats taken from the analyzed population is shown. The corresponding infection doses, expressed as log_10_/mL of cell culture infectious dose 50% (CCID50), are shown.

**Figure 5:**
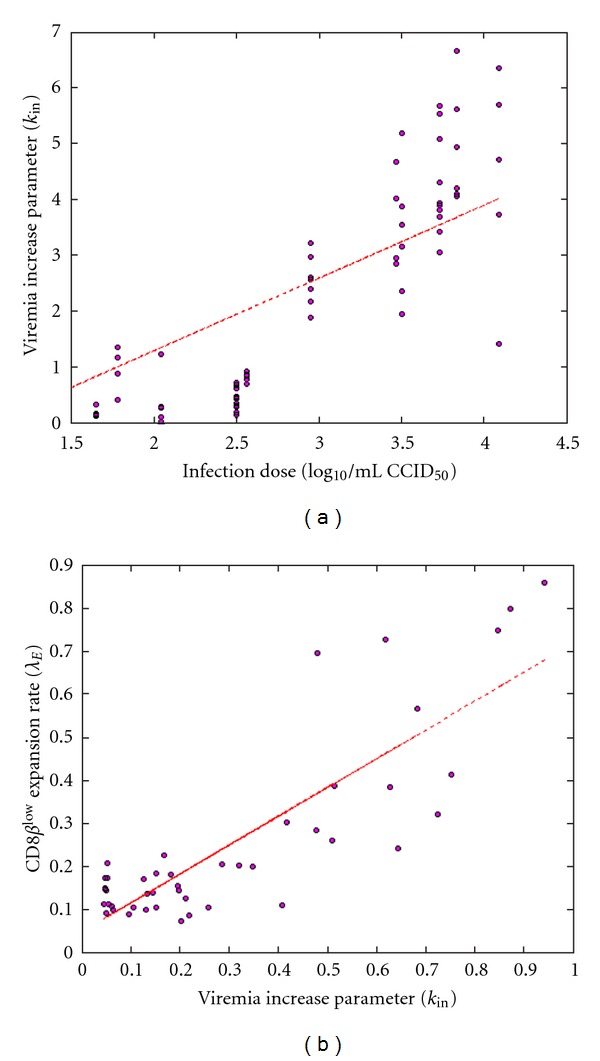
(a) Correlations found between the dose of infection, expressed in log_10_/mL CCID50, and the increase rate of viral load. (b) Resulting correlation between the rate of increase of viral load and CD8*β* ^low^ expansion rate.

Consequently, the value of parameter *k*_in_ increases as the inoculum size increases, ranging from 0.074 weeks^−1^ for the lowest dose to 3.55 weeks^−1^ for the highest dose. This result indicates that the higher the dose of infection, the stronger the increase of viral load in the acute phase. The parameter *k*_out_ was estimated at 0.025 weeks^−1^, and the scaling factor *A*_0_ at 5.56. All parameters were estimated with low-standard errors.  Table 1 presents the parameter estimates of the model as mean values, with standard deviation of random effects or inter-animal variability (IAV).  Figure 3 shows model diagnostics with a visual predictive check, that is, the simulation of the population model with 95% of variability together with the data points (Figure 3(a)) and individual predictions plotted against the actual observations (Figure 3(b)). Correlation between predictions and observations is good (*r* ^2^ = 0.81, *P* < 0.001). In Figure 4, we show individual predictions with a 95% confidence interval around the predictions for six cats taken from the analyzed population and who were challenged with infection doses from 1.65 to 4.09 log_10_/mL. The model correctly predicts the time-evolution of viral load in the individual cats, and a relationship is demonstrated between the dose of infection and the rate of increase of viral load in plasma during the primary stage of infection. This correlation is shown in Figure 5(a), where the estimated values of parameter *k*_in_ for all 102 cats are plotted against the actual values of the infection dose (*r* ^2^ = 0.73, *P* < 0.001).

### 3.2. Modelling CD8*β* ^low^CD62L^−^

In the study presented in [2], the percentage of CD8*β* ^low^ cells is shown to increase in the weeks following infection, eventually reaching a saturation level. We tested several laws, such as a sigmoid function, in an attempt to reproduce this pattern. The best model selected was the Gompertz equation. The model can be written as follows:

(11) $$\begin{array}{c} E=Ke^{-E_{0}e^{-\lambda_{E}t}}, \end{array}$$

where *E* represents the percentage of CD8*β* ^low^ cells, and the parameter *E*_0_ is involved in the expression of the percentage of CD8*β* ^low^ cells at time 0. More precisely, we set *E*(*t* = 0) = *Ke* ^−*E*_0_^. *λ*_*E*_ is a constant term determining the expansion rate and *K* is the maximal percentage of CD8*β* ^low^ cells. The larger the parameter *λ*_*E*_, the sharper the expansion.  Figure 2(b) shows a schematic view of the model and highlights the impact of a change in the value of *λ*_*E*_ on the shape of the curve. Inter-animal variability in the model parameters (*E*_0_, *λ*_*E*_, *K*) was assumed to be lognormal; the dose of infection and virus strain were evaluated as before as covariates. Only the dose of infection, expressed as log_10_/mL of CCID50, was finally successfully integrated into the constant expansion rate of CD8*β* ^low^. With this covariate integration, the objective function was reduced by 33 points, and the variability on the *λ*_*E*_ parameter decreased by 44%.

**Figure 6:**
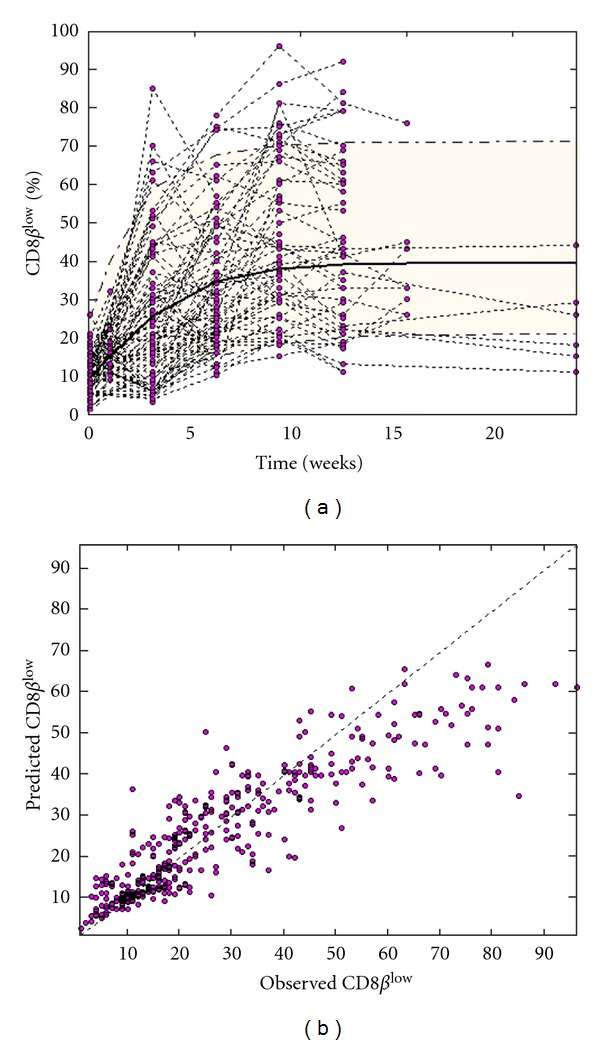
CD8*β* ^low^ model diagnostics. (a): Visual predictive check. (b): Individual predictions versus the actual observations.

The mean value of the maximal percentage (*K*) was estimated at 39.4%. The constant rate of CD8*β* ^low^ expansion increases as the dose increases, ranging from 0.08 weeks^−1^ for the lowest dose to 1.62 weeks^−1^ for the highest dose. This range is very similar to the range of the rate of increase of viral load. Consequently, we observe that the higher the dose of infection, the stronger the expansion of CD8*β* ^low^. Notably, we observe a linear relationship between the rate of expansion of the CD8*β* ^low^ population and the rate of increase of viral load (Figure 5(a)).  Figure 6, similarly to Figure 3, shows model diagnostics with a visual predictive check (Figure 6(a)) and individual predictions plotted against actual observations (Figure 6(b)). Correlations between predictions and observations are fairly good (*r* ^2^ = 0.80, *P* < 0.001), although the highest observations seem to be underestimated by the model. In fact, the proposed model is able to reproduce only the expansion of the CD8*β* ^low^ percentage, whereas in many cases the highest observed CD8*β* ^low^ percentages were followed by lower percentages at subsequent time points (see Figure 6(a)). The latter observation might be attributable to technical variability in performing the laboratory measurements or to fluctuations around a saturation point. 

## 4. Discussion

FIV is a major pathogen affecting cats and is recognized as a relevant model for the study of HIV infection. In particular, during the primary infection phase, the clinical signs and virus localization in FIV-infected cats have been shown to be similar to those observed in HIV infection [1]. The study of primary HIV infection is likely to shed new light on the development of the disease, as a relationship has been shown to exist between the characteristics of acute-stage HIV infection and progression to death due to AIDS [19, 20]. As primary infection in HIV might be difficult to document, the study of the early phase of FIV infection could be an alternative means of gaining insights into HIV that might contribute to the design of new efficient therapy.

In addition to being a valuable model for HIV, FIV on its own constitutes an important research interest. As a result of the growing prevalence and severity of FIV infection, an effective FIV vaccine is greatly needed in veterinary medicine [2]. The issues that researchers have faced in the process of FIV vaccine development are similar to those encountered for HIV, and it is believed that effective vaccines against HIV and against FIV will elicit cellular immune responses [21–24]. 

We performed a longitudinal analysis of important markers of FIV—that is, viral load and CD8*β* ^low^ percentage—in cats undergoing primary infection. The analysis was carried out retrospectively, using data from cats that were infected in an experimental protocol.

This analysis led us to propose two phenomenological models that correctly reproduced the time-evolution of CD8*β* ^low^ percentage and viremia during primary FIV infection in cats. These simple models allowed us to integrate, at the level of the parameters, the intersubject variability that often characterizes preclinical and clinical data. 

Expansion of CD8*β* ^low^ percentage was modelled with a Gompertz law, and viremia was modelled using two exponential laws to reproduce the initial burst of viral load followed by decay. All model parameters were estimated with low-standard errors, and, as expected, variability was elevated for some of the parameters. Even if the models are phenomenological, some of the parameters, and in particular the rate of expansion of the CD8*β* ^low^ population and viral load, can be easily related to the shapes of the curves (see Figure 2 for illustration), and so can be easily interpreted. The dose of infection, expressed as log_10_/mL of CCID50, was found to be a relevant covariate of the rate of expansion of the CD8*β* ^low^ population and the rate of increase of viral load; this covariate explains a large part (up to 30%) of the inter-animal variability on the distribution of these two parameters. Finally, the rate of expansion of viral load and the rate of expansion of CD8*β* ^low^ percentage were observed to be correlated (*r* ^2^ = 0.73, *P* < 0.001; see Figure 5(b)). Obviously, this correlation does not provide any clues regarding the mechanism of action of CD8*β* ^low^ or the relationship between the CD8^+^ T-cell subpopulation and viremia, but it reinforces the prevalent hypothesis that CD8*β* ^low^ percentage is a relevant marker of FIV progression. 

The results we obtained with the proposed models may provide insight into the time course of viremia or viral load and the size of the CD8*β* ^low^ population following infection. Our study points to phenomenological models as a potentially valuable complement to the numerous mechanistic models used to study HIV infection and AIDS progression. For example, researchers have identified a linear relationship between a patient's viral load, taken as the average of all the patient's viral load measurements (allegedly compatible with the concept of a viral set point), and his or her survival time [25, 26]. Our study provides evidence that disease progression in patients can be well described by a simple phenomenological model that does not rely on any biological assumptions. The dynamic approach we adopted here could provide insights into the link between viremia and patient survival [26]. Indeed, the analysis of the time course of viral load might be a better predictor of survival than the average viral load parameter used by Arnaout et al. [25].

## 5. Summary

Cats infected with the feline immunodeficiency virus (FIV) develop an acquired immunodeficiency syndrome (AIDS), similarly to humans infected with HIV. FIV infection causes an acute viremia, which decreases after several weeks, and the appearance of a subpopulation of activated CD8^+^ T cells that we refer to as CD8*β* ^low^ cells. The expansion of this activated T-cell population is recognized as an important marker of FIV infection and disease. Characterization of the CD8*β* ^low^ population's complex pattern of expansion, including its correlation with other disease markers such as viral load, is likely to increase researchers' understanding of FIV infection and AIDS pathogenesis. We propose two simple and independent mathematical equations to analyze the time-evolution of CD8*β* ^low^ population size and of viral load during primary infection in cats with FIV. We develop the models using a population approach and mixed-effects regression techniques, based on repeated measurements in more than 100 cats infected with FIV.

## Figures and Tables

**Table 1:** 

Parameters	Description	Mean value (SE)	IAV	*η*-shrinkage
Viral load				

*k*_in_	Increase rate of viral load	0.06 week^−1^ (21%)	97%	21%
β_*k*_in__	Covariate (cell line) on parameter *k*_in_ (exponential formulation)	1.01 (12%)	—	
*k*_el_	Decay rate of viral load	0.02 week^−1^ (9%)	46%	66%
*A*_0_	Scaling parameter	5.56 (2%)	35%	26%
*a*_*V*_	Parameter of the error residual model (constant formulation)	0.56 (5%)	—	

CD8*β* ^low^				

*λ*_*E*_	Expansion rate of CD8*β* ^low^	0.07 week^−1^ (28%)	74%	35%
*β*_*λ*_*E*__	Covariate (cell line) on parameter *λ*_*E*_ (exponential formulation)	0.77 (17%)	—	
*K*	Maximal CD8*β* ^low^ percentage	39.4 (6%)	56%	23%
*E*_0_	Scaling parameter	1.42 (6%)	54%	31%
*a*_*E*_	Parameter of the error residual model (exponential formulation)	0.41 (5%)	—	
